# Supplementary figures and images for: Analysis of Covid-19 Data for Eight European Countries and the United Kingdom Using a Simplified SIR Model
Source: Res Sq. 2020 Oct 29:rs.3.rs-97697. Preprint. [Version 1] doi: 10.21203/rs.3.rs-97697/v1 (PMC7605556; doi:10.21203/rs.3.rs-97697/v1)

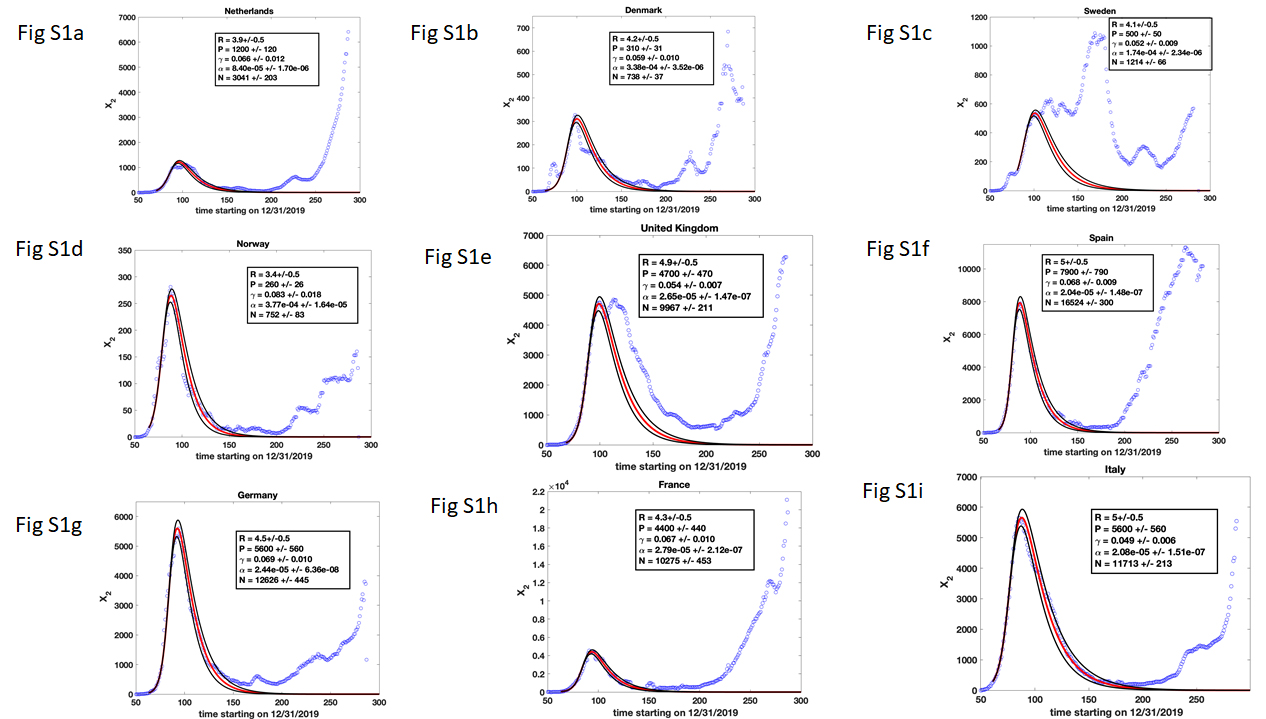

Supplement: Supplement — ary Figure S1: Parameter fits for the number of daily cases plotted on a linear scale: These plots are the same as those in Figure 2 except that the y axis is shown in a linear scale, rather than a log scale. This is to show the accuracy of the fits which are shown as solid lines. Blue circles are observed data for X2(t), the number of cases per day. Solid lines are fits obtained by solving (8) and (9) using the ODE solver ode45 in Matlab. The values of the parameters are shown in the insets and represent the fit shown as the solid red line. The method used for the fits was to find γ(R − 1) from the exponential rise in X2 at early times (Appendix A), estimate the peak value P of X2 (which gives the value of N using (15)) and find an R value that best fits the data (red solid line). The black lines represent solver results for R varying by 0.5 from the best fit value. [file 7b953da04ecfb84fcc253e11.JPG]
