# Supplementary material for: Analysis of Covid-19 Data for Eight European Countries and the United Kingdom Using a Simplified SIR Model
Source: Res Sq. 2020 Oct 29:rs.3.rs-97697. Preprint. [Version 1] doi: 10.21203/rs.3.rs-97697/v1 (PMC7605556; doi:10.21203/rs.3.rs-97697/v1)
Supplement: Supplement — Table 1: Table showing a summary of all results for all 9 countries. [file a064309b3ee4102abc4edc6f.pdf]

TABLE 1	NETHERLANDS	DENMARK	SWEDEN	NORWAY	UK	SPAIN	GERMANY	FRANCE	ITALY
**Mean Latitude**	52.133	56.26	60.13	60.17	52.36	40.46	51.17	46.23	41.87
**Population Miillions**	17.3	5.81	10.2	5.4	66.65	46.9	83	67	60.4
**Major City**	Amsterdam	Copenhagen	Stockholm	Oslo	London	Madrid	Berlin	Paris	Rome
**Average Temp Feb.2020 (deg F)**	38	34	30.5	28	44.5	46	34.5	42.5	47.5
γ(R-1)	0.19	0.19	0.16	0.2	0.21	0.27	0.24	0.22	0.195
**P**	1,200	310	500	260	4,700	7,900	5600	4,400	5,600
**N**	3,041 +/-203	738 +/-37	1,214+/-66	752 +/-83	9,967 +/-211	16,524 +/-300	12,626 +/-445	10,275+/-453	11,713 +/-213
**R**	3.9 +/-0.5	4.2 +/-0.5	4.1 +/-0.5	3.4 +/-0.5	4.9 +/-0.5	5.0 +/-0.5	4.5 +/-0.5	4.3 +/-0.5	5.0 +/-0.50
αx 100,000	8.40 +/-0.17	33.80 +/-0.37	17.44 +/-0.23	37.7 +/-1.64	2.65 +/-0.02	2.04+/-0.02	2.44 +/-0.06	2.79+/-0.02	2.08 +/-0.02
γ	0.066 +/-0.012	0.059 +/-0.010	0.052 +/-0.009	0.083 +/-0.018	0.054 +/-0.007	0.068 +/-0.009	0.069 +/-0.01	0.067 +/-0.01	0.049 +/-0.006
δ	0.130	0.050	0.180	0.030	0.210	0.110	0.050	0.200	0.140
T_D	5	2	5	15	6	5	12	8	4
$T_L = 1/\gamma$	15.3	16.8	19.4	12.0	18.6	14.8	14.6	15.0	20.5
$T_R = 1/(N\alpha)$	3.9	4.0	4.7	3.5	3.8	3.0	3.2	3.5	4.1
**Actual daily cases at first peak**	1,120	328	532	282	4,751	7,902	5,596	4,482	5,644
**Model Fitted daily cases at first peak**	1,219+/-58	311+/-16	536+/-21	265+/-13	4,716+/-234	7,928+/-393	5,601+/-277	4,410+/-218	5659+/-274
**Date when number of daily cases first peaked after 12/31/2019**	4/5/20	4/9/20	4/10/20	3/28/20	4/8/20	3/29/20	4/2/20	4/3/20	3/28/20
**PD(P) = Predicted Pandemic Duration = Number of days from 12/31/2019 to when Daily Deaths < 5 after first peak in daily cases**	169 +/- 10 days	132 +/- 4 days	179 +/- 10 days	117 +/- 3 days	217 +/- 13 days	180 +/- 10 days	175 +/- 9 days	193 +/- 12 days	209 +/- 15 days
**T_{end}(P) = Predicted date pandemic ends = defined as day Daily Deaths < 5 after first peak in daily cases**	6/18/2020 +/- 10 days	5/11/2020 +/- 4 days	6/28/2020 +/- 10 days	4/26/2020 +/- 3 days	8/5/2020 +/- 13 days	6/29/2020 +/- 10 days	6/24/2020 +/- 9 days	7/12/2020 +/- 12 days	7/28/2020 +/- 15 days
**PD(A) = Actual Pandemic Duration = Number of days from 12/31/2019 to when Daily Deaths < 5 after first peak in daily cases**	168	131	207	119	233	180	195	207	214
**T_{end}(A) = Actual date pandemic ends = defined as day Daily Deaths < 5 after first peak in daily cases**	6/17/20	5/10/20	7/26/20	4/28/20	8/21/20	6/29/20	7/14/20	7/19/20	8/2/20
**Predicted Cases upto T_{end}(P)**	45,015 +/-5,413	10,645+/-1,199	22,799 +/-2,657	7,844+/-881	183,393+/- 20,530	242,705+/-26,975	181,375 +/-20,844	151,738 +/-17,726	238,163 +/-26,426
**Actual Cases upto T_{end}(A)**	48,921	10,306	75,228	7,382	320,301	248,240	198,770	179,059	247,164
**Predicted Deaths upto T_{end}(P)**	5,852 +/- 704	533 +/- 60	4,104 +/- 479	236 +/- 27	38,513 +/- 4,312	26,698 +/- 2,968	9,069 +/- 1,043	30,348 +/- 3,546	33,343 +/- 3,705
**Actual Deaths upto T_{end}(A)**	6,066	515	5,732	192	41,384	28,340	9,062	30,180	35,134
**Predicted Cases/Million population from 12/31/2019 to T_{end}(P)**	2602 +/- 313	1832 +/- 206	2235 +/- 260	1453 +/- 163	2752 +/- 308	5175 +/- 575	2185 +/- 251	2265 +/- 265	3943 +/- 438
**Actual Cases/Million population from 12/31/2019 to T_{end}(A)**	2,828	1,774	7,375	1,367	4,806	5,293	2,395	2,673	4,092
**Predicted CFR (deaths/million) from 12/31/2019 to T_{end}(P)**	338 +/- 41	92 +/- 10	402 +/- 47	44 +/- 5	578 +/- 65	569 +/- 63	109 +/- 13	453 +/- 53	552 +/- 61
**Actual CFR (deaths/million) from 12/31/2019 to T_{end}(A)**	351	89	562	36	621	604	109	450	582
